# Nutritional values and health benefits of dromedary camel meat

**DOI:** 10.1093/af/vfac051

**Published:** 2022-08-12

**Authors:** Isam T Kadim, Issa S Al-Amri, Abdulaziz Y Alkindi, Quazi M I Haq

**Affiliations:** Department of Biological Sciences and Chemistry, College of Arts and Sciences, University of Nizwa, PO Box 33, PC 616, Birkat Al-Mouz, Nizwa, Sultanate of Oman; Department of Biological Sciences and Chemistry, College of Arts and Sciences, University of Nizwa, PO Box 33, PC 616, Birkat Al-Mouz, Nizwa, Sultanate of Oman; Department of Biological Sciences and Chemistry, College of Arts and Sciences, University of Nizwa, PO Box 33, PC 616, Birkat Al-Mouz, Nizwa, Sultanate of Oman; Department of Biological Sciences and Chemistry, College of Arts and Sciences, University of Nizwa, PO Box 33, PC 616, Birkat Al-Mouz, Nizwa, Sultanate of Oman

**Keywords:** bioactive, camel, functional properties, health benefits, nutritive value

**Implications**

- Camel meat products are receiving increased interest on a worldwide scale due to its high functional properties and nutritive values.
- Camel meat products contain many essential nutrients, and some components with potential bioactive properties that could be beneficial for human health and wellbeing.
- The health remunerations of camel meat products are a promising future perspective that can be used as a tool to enhance the value of functional meat.
- Similar to other red meat products, enrichment of camel meat with health-promoting substances are not a new approach, but it can be recommended for human consumption.

## Introduction

The opinion of meat consumers has altered over recent decades from considering meat products simply as a source of essential nutrients to consider meat as a health-promoting supplement (Kadim et al., 2020). Therefore, substantial changes in the global meat products’ market occur with increasing demand for high nutritional values and healthy meat products (Kadim et al., 2018). Health benefits are the main factor influencing consumer demand for any meat products available in the market. A result of interest from the preference shift of consumers is that the functional properties of camel meat products might be considered as an alternative health food. In this respect, a remarkable effort in the meat industry has been directed towards enhancing nutritional values and healthiness of meat products (Decker and Park, 2010). According to Al-Abri and Faye (2019) and Bouhaddaoui et al. (2019), hot environments harmfully affect animals causing heat-stress effects on health, but camels have adapted to produce healthy meat even in the hottest and least favorable environmental conditions. Kadim et al. (2020) stated that opportunities exist to improve the nutritional and potential health features of camel meat products, natural production, or addition of natural functional substances. The function properties of camel meat products may be increased by healthy adding ingredients (Pogorzelska-Nowicka et al., 2018). Camel meat is believed by Somali and Indian people to have remedial effects for different health disorders as hyperacidity, hypertension, or pneumonia (Kurtu, 2004). Applying the advance technology can improve nutritional and health benefits of camel meat products and development of new products. Recently, consumers are interested, and may pay more for meat products supplemented with bioactive compounds. Marketing the high-quality parameters and nutritive values of camel meat products are a promising future perspective that may be implemented to enhance the value of functional camel meat. This article aims to identify the important nutritional and potential health components and focus on future developments of nutritional aspects of camel meat.

## Nutrient Contents

An outline of the nutritional characteristics of camel meat is provided in Table 1 with an indication of why they are important for human nutrition and comments on special aspects with respect to the meat from camels. Details on individual nutrients are provided in subsequent sections. Information on the proximate composition of camel meat and other meats in Table 2 illustrates that, except for fat content, camel meat composition is generally similar to meat from other meat-producing mammals such as cattle, sheep, and deer. Moisture contents in camel meat widely varied (63.0% to 77.7%) with higher values generally being associated with lower fat percentages. Kadim et al. (2006) stated that with age, the moisture contents of the camel meat decreased. Ibrahim et al. (2015) reported that small difference in moisture contents between 3–4- and 6–7-year-old camels across different muscles, while Gheisari et al. (2009) found no such difference in moisture contents between camel meat and meat from other species of similar gender and age. The protein content of camel meat ranges from 17.1% to 22.1% (Table 2), with meat from young camels containing similar protein percentages to those found in young cattle, goat, and lamb meats (Kadim et al., 2008). Some other factors may also affect the fat content of camel meat within similar age groups (Kadim et al. 2006, 2008, 2009a, 2009b).

**Table 1.** Examples of meat characteristics of importance for human nutrition with comments on these for meat from camels. Variation in these characteristics due to factors such as genotype, animal diet, and differences between muscles may often be important but are not considered in this broad overview (Kadim et al., 2020)

Nutritional characteristic	Importance for human diets and presence in meat	Comments regarding camel meat
Protein concentration	Proteins are an essential requirement of the human diet, with meat being an important source for many people.	Protein concentrations are slightly higher in camel meat than for many other meats due to lower fat levels (see Tables 2– 4).
The amino-acid balance within proteins and as free amino acids.	The balance of the 12 essential amino acids in meat is close to estimated requirements for humans.	A similar balance of amino acids is found in camel meat to that of meat from other mammals (see Table 4).
Digestibility and bioavailability^a^ of proteins and amino acids.	Proteins must be digestible and absorbable to be useful. Generally, proteins are high digestible for most meat proteins when cooked with care.	Highly digestible proteins as in other meats (see Section 5).
Bioactivity^b^ of proteins or their breakdown products.	There is increasing evidence that certain short-chain polypeptides from meat proteins have bioactive properties.	Limited research is available, in particular with proteins of camel meat (see Section 5).
Lipid (fat) concentration	Some lipid components are essential for humans, but fat is mainly a source of energy. Some lipids are undesirable nutritionally.	Concentrations are generally low in camel meats (see Table 3).
Fatty-acid proportions in lipid	Some fatty acids in meat are beneficial (e.g., long-chain-n3), while some are undesirable for humans (e.g., certain saturated FAs and some trans fats).	The balance of fatty acids in camel meat is generally good but is dependent on camel diets and levels of fatness (see Table 3).
Minerals	Many minerals are essential for humans with meat being an important source of several key minerals including iron and zinc. Potentially harmful minerals are usually absent.	Similar trend is found in camel meat to other red meats (see Table 5).
Bioavailability of individual minerals.	Important minerals such as iron in meat are more bioavailable than for the same minerals in many other foods.	Expected to mirror the case for other red meats.
Water-soluble vitamins	Required components of human diets, with meat being an important source, especially for vitamin B12. A poor source of vitamin C.	Similar to other red meats based on limited data (see Table 6).
Fat-soluble vitamins	Also required in the human diet, with levels depending to some extent on fat levels in the meat.	Will tend to be at lower levels due to low concentrations of lipid (see Table 6).
Other compounds with possible bioactive properties	A developing area with examples of compounds being evaluated in meat including coenzyme, taurine, lipoic acid, carnitine, carnosine, creatine, growth factors, etc.	Limited information is available, but expected to be similar to other red meats.

^a^Bioavailability: The effectiveness with which components of the diet are taken up an effectively utilized by cells within the body.

^b^Bioactivity: A compound is said to be bioactive if it contributes to human health and wellbeing in some way over and above its effect as a nutrient.

**Table 2.** Chemical composition (g/100 g) of meat of different species

No of animals/Species	Muscle	Moisture	Protein	Fat	Ash	References
Dromedary	LT	73.8	19.0	**6.2**	0.85	
	IS	73.2	18.2	**5.3**	096	
	TB	77.7	17.1	**1.9**	1.00	
	ST	75.4	18.5	**3.1**	0.91	Kadim et al. (2013)
	SM	63.0	22.1	**2.5**	0.93	
	BF	74.3	20.8	**2.5**	1.00	
20 Lama	LT	73.9	23.1	**0.5**	2.40	Cristofanelli et al. (2004)
40 Alpaca	LT	73.6	23.3	**0.5**	2.50	
70 Guanaco	LT	73.9	20.9	**1.0**	1.10	Gonzalez et al. (2004)
6 Beef	LD	70.9	20.0	**5.7**	0.98	Moreira et al. (2003)
17 Beef	BF	72.2	21.1	**6.1**	0.96	Purchase al. (2014)

## Health Benefits

Camel meat products may be marketed as a functional food by identifying the dietetic values and bioactive components with a potential health benefit for consumers (Kadim et al., 2014; Abrhaley and Leta, 2018; Kadim and Sahi, 2018). According to Mollet and Rowlan (2002), although, meat products should satisfy hunger and provide necessary nutrients, it should also improve their health and prevent nutrition-related diseases (Menrad, 2003). Meat consumers prefer to intake healthier meat products without fundamentally changing their eating patterns. Such attitude could contribute to the development of camel meat market.

Researchers found that camel meat products are rich in essential amino acids and minerals, vitamins, bioactive components (carnosine, anserine, glutathione), and essential fatty acids (Kadim et al. 2008, 2010, 2013). Biesalski (2005) stated that consumer’s health problems including obesity, high triglycerids, and high cholesterol are linked with increasing consumption of animal products, therefore, total dietary fat intake should be reduced (Schönfeldt and Gibson, 2008). According to the recommendation of WHO (2003), total fat, saturated fatty acids (SFAs), essential omega-3 polyunsaturated fatty acids (PUFAs) should contribute <15–30%, <10%, and <1–2% of the total energy intake, respectively. In general, the low cholesterol and fat contents of camel meat products might support its dietetic advantage as a better alternative to the high fat content meat products (Bin Saeed et al., 2005; Kadim et al., 2008; 2014; Kadim and Sahi, 2018).

## Nutritional Values

Camel meat products contain high nutritional value, micronutrients important for human health, and essential omega-3 polyunsaturated fats (Kadim et al., 2008, Kadim et al., 2018; Ibrahim et al., 2018) (Table 1). Although, camel meat products significantly tend to have low fat content (Williams et al., 2007), the nutritional values will vary depending on breed, feeding regimen, age, season, and meat cut. Researchers reported that camel meat products contained relatively low fat content with high unsaturated fatty acids (UFAs) and low cholesterol levels, and is rich in protein and many essential vitamins and minerals (Kadim et al., 2013).

## Fatty Acids Profile

Fatty acid composition of meat product is of great concern because of its important effects on consumers’ health (Blasbalg et al., 2011). The protein content of the camel meat is significantly greater and intramuscular fat is significantly lower than veal (Kadim et al., 2008). Decreasing of fat intake is important to potentially reduce obesity, and hypercholesterolemia (Chan, 2004). An epidemiological study by Siri-Tarino et al. (2010) supported the association between SFAs and cardiovascular disease and recommended to reduce intake of SFAs and increasing consumption of omega-3. In this respect, Kadim et al. (2008) found that camel meat products containing relatively high level of polyunsaturated fat acids and low cholesterol levels, which it can be recommended to reduce obesity. Table 3 supported the above conclusion by showing that intramuscular fat from camel meat contained lower total SFAs, higher UFAs and PUFAs than beef cattle meat. Furthermore, Mozaffarian et al. (2010) stated that the high contribution of saturated fat in consumers’ diets connected with high cholesterol intake is assumed to be linked with the incidence of diet-related diseases including coronary diseases. Therefore, to lower meat fat intake, camel meat product can be considered a suitable product due to low intramuscular fat content (Table 3). On average 45.0% of total fatty acids is SFAs in the camel muscle and approximately ½ of the SFA is palmitic acid (16:0), and 1/3 is stearic acid (18:0). The predominant fatty acids in dromedary camel meat were in the same order: oleic (33.5%), palmitic (28.5%), 357 stearic (19.3%), and palmitoleic acid (6.3%) with a percentage of polyunsaturated of 5.6% only (Kadim et al., 2011). On the other hand, PUFAs (PUFA) ranged from 7.2% to 12.8% of total fatty acids. In this respect, twice the amount of oleic (C18:1) and less than ½ the amount of linoleic acid (C18:2) were found in camel meat products (Al-Bachir and Zeinou, 2009; Kadim et al*.,* 2011). The main PUFAs in camel meat products were linoleic acid (C18:2n6c) and arachidonic acid (C20:4n6). The amount of PUFA in camel meat product (11.2%), that is higher than beef (8.8%) and lower than deer (31.4%) (Sinclair et al., 1982). The ratio of C18:2n6c and C18:2 in camel meat product is 13:9 whereas it is higher in meat of cattle, sheep, or goat (2.0, 2.4, and 2.8, respectively) (Sinclair et al., 1982).

**Table 3.** Fat, cholesterol content, and fatty acid composition determined in various species meat

	Fatty acid%										
Species/Muscle	C14:0	C16:0	C18:0	C18:1	C18:2	C18:3	C20:4	TSFA	TUFA	TPUFA	
20 Lama LT	4.09	24.8	21.5	35.8	3.13	0.82	1.78	**50.3**	**42.5**	**7.2**	Polidori et al., 2007
Dromedary IS	7.62	27.6	8.79	25.0	7.14	0.64	2.81	**51.8**	**-**	**11.4**	
Dromedary TB	7.78	27.3	8.90	26.3	7.83	0.43	2.72	**50.8**	**48.2**	**11.6**	
Dromedary LT	7.16	26.9	9.82	26.2	7.11	0.59	2.84	**51.3**	**49.2**	**11.5**	
Dromedary ST	7.24	25.1	8.71	26.4	7.79	0.62	2.83	**48.6**	**48.7**	**12.3**	Kadim et al. (2013)
Dromedary SM	7.48	26.5	8.37	26.8	7.98	0.54	2.55	**49.9**	**50.4**	**11.9**	
Dromedary BF	7.83	26.2	8.02	26.9	7.94	0.54	3.51	**48.9**	**50.2**	**12.8**	
Dromedary LT	8.88	26.1	12.0	25.9	12.7	1.22	-	**48.9**	**51.1**	**15..4**	
Dromedary BF	8.78	25.4	12.2	25.2	14.2	1.11	-	**48.6**	**51.4**	**16.6**	Ibrahim et al. (2017)
Dromedary ST	8.91	26.4	12.6	23.4	14.0	1.64	-	**50.0**	**50.0**	**15.9**	
Dromedary SM	8.25	27.2	11.9	25.8	13.6	1.21	-	**49.2**	**50.8**	**16.2**	
Dromedary TB	7.60	27.5	8.69	21.5	7.14	0.65	-	**-**	**-**	-	
Dromedary IS	7.35	25.3	8.76	26.3	7.83	0.64	-	**-**	**-**	**-**	
Dromedary BF	7.13	27.4	9.80	26.6	7.10	0.66	-	**-**	**-**	**-**	Raiymbek et al. (2019)
Dromedary ST	7.46	26.8	8.40	27.1	7.66	0.56	-	**-**	**-**	**-**	
Dromedary LT	7.81	27.7	8.18	26.8	7.68	0.54	-	**-**	**-**	**-**	
Dromedary SM	7.84	27.3	8.95	21.3	7.88	0.44	-	**-**	**-**	**-**	
Beef cattle LT	3.25	33.9	16.6	36.9	3.86	0.86	-	**54.1**	**40.5**	**5.44**	Brugiapaglia et al. (2007)

The intramuscular fat from camel meat products contained 50 mg/100 g cholesterol level, which is lower than in lamb and beef meat fats (196 and 206 mg/100 g fresh weight, respectively) (Abu-Tarboush and Dawood, 1993). Similar conclusions were supported by Kadim et al. (2008) and Raiymbek et al. (2019).

## Amino Acid Profile

**Figure 1.**
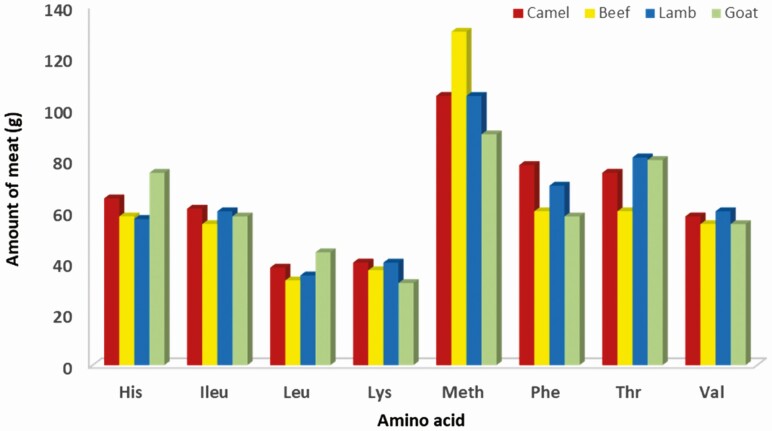
The amounts of meat from different species required to supply the daily requirements of essential amino acids for adults (70 kg body weight) based on mean nitrogen requirement of 105mg nitrogen/kg/day (0.66g protein/kg/day) recommended by WHO (2002).

Camel meat products contain similar essential amino acid composition to beef, lamb, and goat meat products (Table 4) with higher lysine and methionine percentage than ostrich meat products (Al-Shabib and Abu-Tarboush, 2004). In general, meat is a rich source of protein and various bioactive compounds that impart several health benefits (Baba et al., 2021). Amino acids and bioactive compounds in meat and connective tissue contribute to prevent sarcopenia and maintain blood pressure through ACE inhibitory components (Baba et al., 2021). According to Casey (1993), it has been stated that the quality of animal meat protein lies in the availability of lysine and leucine in proportions required by consumers. In this respect, Table 4 shows that the amount of camel meat required to supply the daily requirements of essential amino acids for adults is compatible to lamb meat. The lysine and leucine requirements for an adult man (70 kg) are 2.1 and 2.7 g/day (FAO/WHO/UNU, 2007), respectively. Therefore, 150 g of lean camel meat will cover the daily requirement for lysine and leucine. Furthermore, 100–200 g of camel meat would be an excellent source of high-quality proteins as it contains major essential amino acids in an appropriate ratio (Institute of Medicine, Food and Nutrition, 2002). The amount of camel meat required to supply the daily requirements of essential amino acids for adult (70 kg body weight) is similar to that from lamb, beef, and goat (Figure 1).

**Table 4.** Reported composition of the amino acids in camel meat

	Essential Amino acids																	
Factor	His	Ileu	Leu	Lys	Met	Phe	Thr	Trp	Val	Ala	Arg	Asp	Glu	Gly	Pro	Ser	Tyr	Reference
**Dromedary camel**																		
LT	4.0	5.8	8.1	8.3	3.6	6.9	4.9	0.8	5.3	4.8	6.8	9.9	16.1	2.24	–	3.5	3.8	
BF	3.8	5.1	6.9	8.9	3.7	4.9	4.8	0.8	4.6	4.8	6.9	9.9	16.9	1.82	–	3.9	3.8	Ibrahim et al. (2017)
ST	4.1	5.7	7.0	7.9	3.6	5.0	4.9	0.7	5.1	4.9	7.0	9.9	16.7	2.39	–	3.8	3.8	
SM	3.8	5.1	6.9	8.7	3.7	5.0	5.0	0.7	4.9	4.9	7.0	10	16.9	2.11	–	3.9	3.9	
LT^6^	4.4	4.7	8.3	9.4	2.9	4.3	4.5	–	5.6	6.5	6.6	9.3	15.9	4.3	3.9	3.6	3.5	Kadim et al. (2011) *
Loin	3.4	4.2	7.1	9.1	1.6	5.6	4.8	1.6	4.7	–	–	–	–	–	–	–	–	Al-Shabib andAbu-Tarboush (2004)
Leg	3.4	4.3	8.4	9.1	1.3	5.5	4.8	1.9	4.6	–	–	–	–	–	–	–	–	
Chuck	4.7	5.3	8.6	8.4	2.6	4.1	4.2	0.5	4.9	6.3	7.5	9.3	17.1	6.0	5.4	3.5	3.0	Dawood and Alkanhal (1995)
Ribeye	4.3	5.4	8.3	8.6	2.2	4.4	4.7	0.7	5.3	6.2	7.1	9.3	17.3	5.9	4.9	3.8	3.4	
Leg	4.5	4.9	8.3	8.3	2.5	4.2	4.2	0.6	5.4	6.3	7.5	8.6	16.4	5.9	5.9	3.6	3.3	
Camel	5.6	5.9	9.5	8.9	3.5	4.7	4.8	–	6.3	3.9	7.1	10.8	18.6	6.1	3.9	3.2	3.8	Elgasim and Alkanhal (1992)
Beef	6.2	6.5	10.7	9.1	2.7	5.7	5.5	–	6.6	7.7	7.1	10.8	16.5	6.2	4.5	4.2	4.1	
Lamb	5.9	5.8	9.6	8.5	3.3	4.9	4.2	–	5.9	6.7	6.9	10.3	17.9	5.5	3.8	2.9	3.5	
Goat	4.7	6.0	7.9	11	3.9	6.5	4.4	–	6.8	4.7	7.1	10.8	15.6	5.2	3.8	3.6	5.9	
Camel	3.4	4.3	7.7	9.1	1.4	5.5	4.8	1.8	4.7	6.5	6.9	9.7	17.0	6.2	–	4.3	3.3	Al-Shabib and Abu-Tarboush (2004)
Ostrich	2.8	3.8	7.4	4.3	0.5	4.9	4.2	1.8	3.8	5.6	5.9	8.3	15.4	4.5	–	3.7	2.8	

^*^Calculated from mg/100 DM values using the average DM and protein contents.

^1^IS: Infraspinatus,

^2^SM: Semimembranosus,

^3^TB: Triceps brachii,

^4^ST: semitendinosus,

^5^BF: Biceps femoris,

^6^LT: longissimus thoraces,

The most abundant essential amino acids in camel meat products were lysine, then leucine, methionine, isoleucine, threonine, and phenylalanine (Kadim et al, 2011; Raiymbek et al, 2015; Abdelhadi et al, 2017). Leucine and lysine were ranged from 6.8%–9.9%, and 8.1%–9.9% of protein, respectively (Table 4). The essential amino acid profiles in camel loin and leg meat cuts differed by >2.1% with the exception of leucine, methionine, and tryptophan, which differed by 18.5%, 25.4%, and 14.6%, respectively (Al-Shabib and Abu-Tarboush, 2004), while the differences between chuck, ribeye, and leg meat cuts reached >4.2% (Dawood and Alkanhal, 1995). In general, differences in essential amino acids between different cuts ranged between 0.6% and 166.7% (Elgasim and Alkanhal, 1992; Dawood and Alkanhal, 1995; Al-Shabib and Abu-Tarboush, 2004). However, tryptophane level in camel meat cuts was lower than in other meat cuts (Dawood and Alkanhal, 1995). Compared to other red meat, camel meat has the higher essential amino-acid index (Rayimbek et al., 2015) emphasizing its high proteinic value. The non-essential amino acid profiles slightly varied also between camel meat cuts. The range of glutamic acids, aspartic, arginine, and proline were from 15.2%–18.6%, 8.6%–10.8%, 6.6%–7.8%, and 3.9%–5.9%, respectively (Table 4). The range of serine, tyrosine, and alanine were 3.1–4.1, 3.0–4.2, and alanine 3.9–6.4 g/100 g protein, respectively. Moreover, camel meat may be a better source of non-essential amino acids than beef, lamb, and goat meats (Table 4).

## Mineral Profile

Camel meat products are regarded as good sources of minerals for consumers due to natural feed eaten by camels (Kadim et al., 2020). Minerals include those required as essential elements for growth and health, and those that are toxic, but the latter will not be considered here.

Potassium is one of the major elements in camel meat products (105 mg/100 g fresh weight), while sodium was in the range of 67.1–312 mg/100 g (Table 5). Phosphorus is the second most abundant element in camel meat (105.6–199.0 mg/100 g fresh weight). Sulfur content was in the range of 54.99–136.57 mg/100 g fresh weight (Table 5). According to Moshfegh et al. (2009), the Recommended Dietary Allowance (RDA) of Ca is 1000–1200 mg/day and for Mg 320–420 mg/day. The average content of Ca in different species ranges from 5.9 mg/100 g (camel) to 17 mg (pork), while for magnesium it ranges from 12.9 mg/100 g (camel) to 25 mg (turkey) (Kadim et al., 2009a; Peter, 2017). Ca content of camel meats cuts ranged from 19% to 27% (Dawood and Alkanhal, 1995; Rashed, 2002).

**Table 5.** Mineral concentrations in camel meat (mg/100 g fresh weight)

				Mineral^1^								
Factor	Ca	Cu	Fe	K	Mg	Mn	Sl	Na	P	S	Zn	
Rump	–	0.12	2.5	–	–	–	–	–	–	–	–	
Loin	10.2	0.16	44.0	446	28.0	0.16	–	189	–	–	66.0	
leg	9.8	0.26	50.5	548	42.5	0.19	–	313	–	–	85.5	
Chuck	11.5	–	3.2	249	17.4	–	–	73.5	–	–	3.7	Dawood and Alkanhal (1995)
Loin	8.1	–	2.9	231	16.3	–	–	67.1	–	–	3.7	
limb	10.3	–	3.4	251	17.1	–	–	69.7	–	–	3.9	
Shoulder	5.1	0.07	1.2	357	20.6	0.01	–	69.1	196	56.1	3.5	El-Faer et al. (1991)
Thigh	5.4	0.09	1.4	361	21.0	0.01	–	70.4	199	55.0	3.1	
Ribs	4.7	0.07	1.2	324	18.5	0.01	–	84.1	181	58.0	3.9	
Neck	5.6	0.09	1.4	338	18.5	0.01	–	87.3	181	64.4	4.8	
Camel	5.9	–	–	193	12.9	–	–	45.3	105	–	–	Kadim et al. (2009a)
Beef	6.2	–	–	416	20.5	–	–	51.0	162	–	–	
Camel	4.9	0.04	1.9	228	17.7	0.01	–	47.9	–	–	3.2	Elgasim & Alkanhal (1992)
Camel												
LT	13.3	4.11	3.25	797	37.1	0.15	–	149	352	–	5.11	
ST	14.1	4.41	3.55	751	34.9	0.14	–	139	355	–	4.98	Ibrahim et al. (2017)
SM	14.4	4.55	3.89	778	35.6	0.13	–	141	389	–	5.49	
BF	13.6	4.99	3.22	759	35.9	0.14	–	141	393	–	5.58	
Llama	11.6	–	3.3	447	28.4	106	–	–	379	–	4.4	Polidori et al. (2007)
Alpaca	8.8	–	3.0	412	23.1	92.8	–	–	338	–	3.9	
Beef BF	4.7	–	1.91	325	20.7	0.02	–	50.4	181	–	3.7	Purchas et al. (2014)
Lamb LT	9.7	–	–	328	22.2		4.5	71.8	187	–	3.02	

^1^Mineral: Ca: Calcium; Cu: Copper; Fe: Iron; K: Potassium; Mg: magnesium; Mn. Manganese; Sl Selnium, Na: Sodium; P; Phosphorus; S; Sulfate Zn: Zinc

Iron is one of the key minerals in human nutrition because of its physiological functions including oxygen transport, synthesis of enzymes, energy production, and regulation of immune functions (Radlowski and Johnson, 2013). Iron is also playing a significant rule in the brain development of the fetus and further in maintenance of neural connection. Semi-intensive feeding systems of camels may increase its meat with this mineral. Camel meat products could be perceived as functional foods due to the amounts of iron they contain. Camel meat can enhance muscle functions, nerve transmission, intracellular transmission, vascular contraction, and vasodilation (Beto, 2015). Camel meat products contain 10.41–21.03 mg/100 g fresh weight (Kadim et al., 2009a) which is a cofactor for many enzyme systems, takes part in energy metabolism and the synthesis of proteins and nucleotides (De Baaij et al., 2015). Selenium element is important for human health because it is a part of selenoproteins and it regulates many physiological functions (Pogorzelska-Nowicka et al., 2018). It plays an important role in antioxidative defense, immune system regulation, metabolism of thyroid hormones, male reproduction, prevention of pre-eclampsia, diabetes mellitus, cardiovascular diseases, and cancer (Riaz and Mehmood, 2012). Zinc has catalytic and over 100 enzymes are zinc-dependent, and it is important in protein and cell membrane maintenance and regulatory gene expression functions in cells (Roohani et al., 2013). Red meat is an important source of Zn and camel meat contains about 3.07 to 4.10 mg/100 g fresh weight. Zinc is an essential trace element for human health, with over 100 enzymes being zinc-dependent (Kadim et al 2020). Zn can decrease fat oxidation, reduced cooking loss, increased crude protein content, and increased total antioxidant capacity in meat were observed (Yang et al., 2016).

## Vitamin Profile

Due to low fat content of camel meat, fat-soluble vitamins as vit A, are in low quantity compared to other species. Raiymbek et al. (2018) reported that camel meat contained 9.97–10.5 μg/100 g vit A. Vit E possesses antioxidant ability to break the chain reactions of free radical formation (Pearce and Jacob, 2004) and react against oxidation of the plasma lipoproteins and PUFA components of cell membranes (Horba et al., 2016). Deficiency of vit D may cause cardiovascular disease, type 1 diabetes, cancer, hypertension, rheumatoid arthritis, autoimmune conditions, and Parkinson’s disease. Human daily required is around 10–20 µg/day (400–800 IU/day) assuming little or no exposure to sun, while it is shown that the actual intake is usually only about 3–7 µg/day (120–280 IU/day). Consumption of sufficient amounts of B-group vitamins is essential for proper functioning of human body and particularly important are folate (B9) and vitamin B_12_ (Kadim et al., 2013). The B-vitamin complex in camel meat products is varied in quantities from a few micrograms to several milligrams per 100 g (Table 6). The range of vit B_1_ in camel meat from 0.08 to 0.0 mg/100 g determined in camel muscles (Table 6). The thiamin levels in camel muscle products (0.09 mg/100 g) were higher than beef (0.5 mg/100 g) lamb (0.06 mg/100 g), rabbit (0.05 mg/100 g), chicken (0.04 mg/100 g), and Turkey meats (0.02 mg/100 g) (Lombardi-Boccia et al (2005). Meat is usually contributed 77% of the vit B_12_ in the diet (Karmas, 1988). Fifty grams of camel meat product contain 2.38g/100 g vit B_12_, that represent 118% of the human RDA for vitamin B_12_. The average camel meat contained 4.75μg/100 g vit B_12_, which provides ample amounts of this vitamin. The camel meat had higher vit B_12_ than sheep (0.25 mg/100 g) and veal meats (0.18 mg/100 g). Vit B_6_ is related to protein content of the diet. It is also necessary for the formation of hemoglobin (Henderson et al, 2003). The vit B_6_ concentration in camel ranged from 0.61 to 0.67 mg/100 g which are higher than 0.35 to 0.49 mg/100 g for pork meat, turkey meat (0.42 mg/100 g), chicken meat (0.53 mg/100 g), and fish (0.34 mg/100 g) (Sauberlich et al, 1982). An average serving of camel meat (200 g) provides 80% of the RDA for vit B_6_ for the young adult male. Pantothenic acid plays a key role in energy metabolism. The range values of pantothenic acid in camel muscles were 0.82–0.89 mg/100 g. Riboflavin is necessary for normal growth and helps maintain the integrity of mucous membranes, skin, eyes, and nervous system (Henderson et al, 2003). Riboflavin is found in red meat and 15% of the average daily intake in human is derived from meat and meat products.

**Table 6.** Vitamins of camel meat

						Vitamin^1^					
Species	Muscle	B1	B2	B3	B5	B6	B12	A	D	E	
Dromedary	LT	0.11	0.23		0.78	0.59	4.64	10.5		0.85	IIbrahim et al. (2018)
	ST	0.08	0.22		0.76	0.61	4.77	11.2		0.92	
	SM	0.09	0.26		0.72	0.61	4.68	10.1		0.86	
	BF	0.09	0.26		0.77	0.62	4.69	9.99		0.83	
Beef	BF	0.05	0.10	3.49	0.39	0.27	1.69	9.38	0.15	0.45	Purchas et al. (2014)
Lamb	LT	0.10	0.16	5.13	0.50	0.15	1.85	4.69	0.04	0.29	

^1^B1: Thiamine (mg/100 g), B2: Riboflavin (mg/100r), B3: Niacin (mg/100 g), B5: Pantothenic acid (mg/100 g), B6: Pyridoxine (mg/100 g), B12: Cyanocobalamin (μg/100 g), A: Retinol (μg/100 g), D: D3 Cholecalciferol (μg/100 g), E: Alpha-Tocopherol (E) (mg/100 g)

## Bioactive Compounds

**Figure 2.**
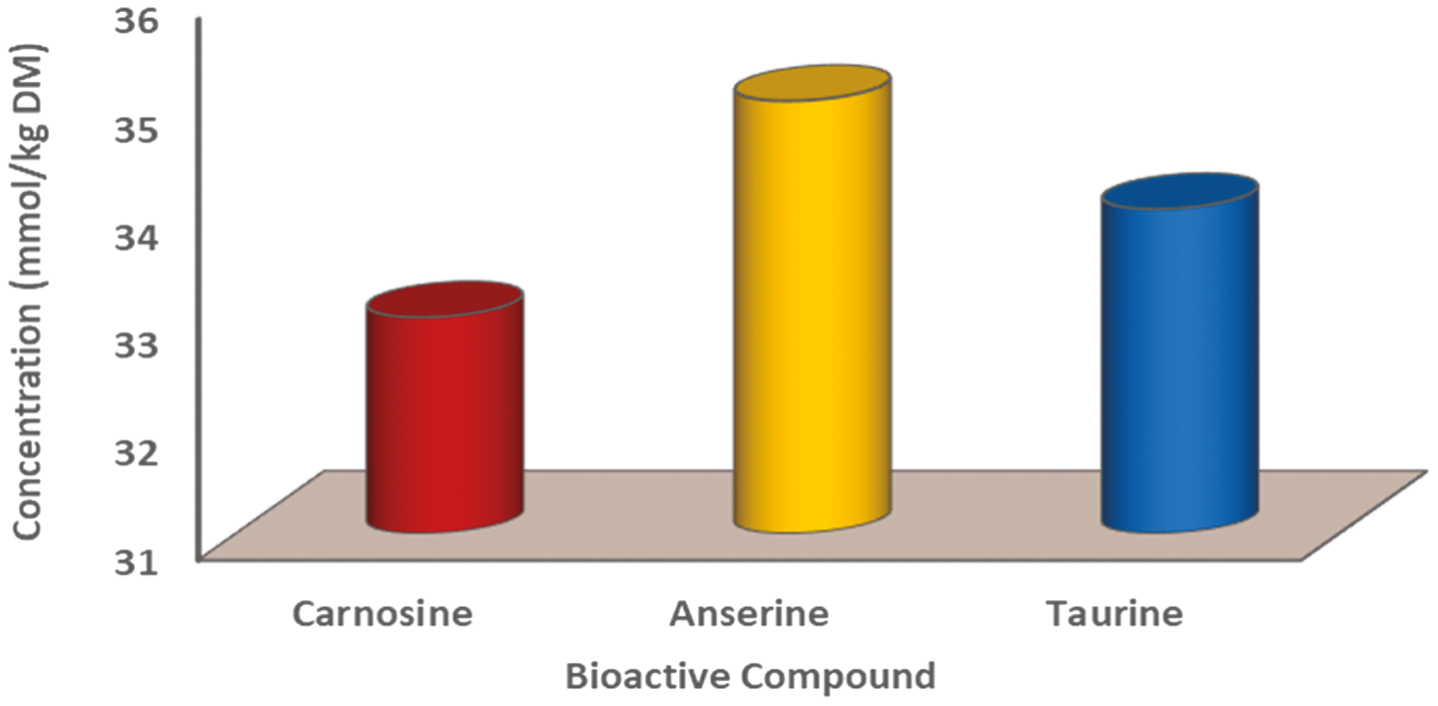
Average concentrations of carnosine, anserine, and taurine in camel middle gluteal muscle. From Dunnett et al. (1997) and Dunnett and Harris (1997).

Several bioactive compounds have been investigated in meat that are nutritionally important and can potentially be useful in marketing meat products (Arihara, 2006). Carnosine (β-alanyl-L-histidine) and its derivative anserine (β-alanyl-1-Methyl-L-histidine) are important dipeptides which are found in high concentrations in the meat products (Tomonaga et al., 2006). The same authors stated that their function is antioxidants and putative neurotransmitters in the brain (Tomonaga et al., 2006). High concentrations of about 365 and 400 mg/100 g have been reported in beef and lamb, respectively (Purchas et al., 2004) and in red deer, 290 and 329 mg/100 g (Purchas et al., 2010). The average levels of carnosine and anserine in camel meat was 181.7 mg/100 g and 268.6 mg/100 g fresh weight, respectively (Dunnett et al., 1997; Dunnett and Harris, 1997). The values in mmol/kg DM are reported in Figure 2.

**Figure 3.**
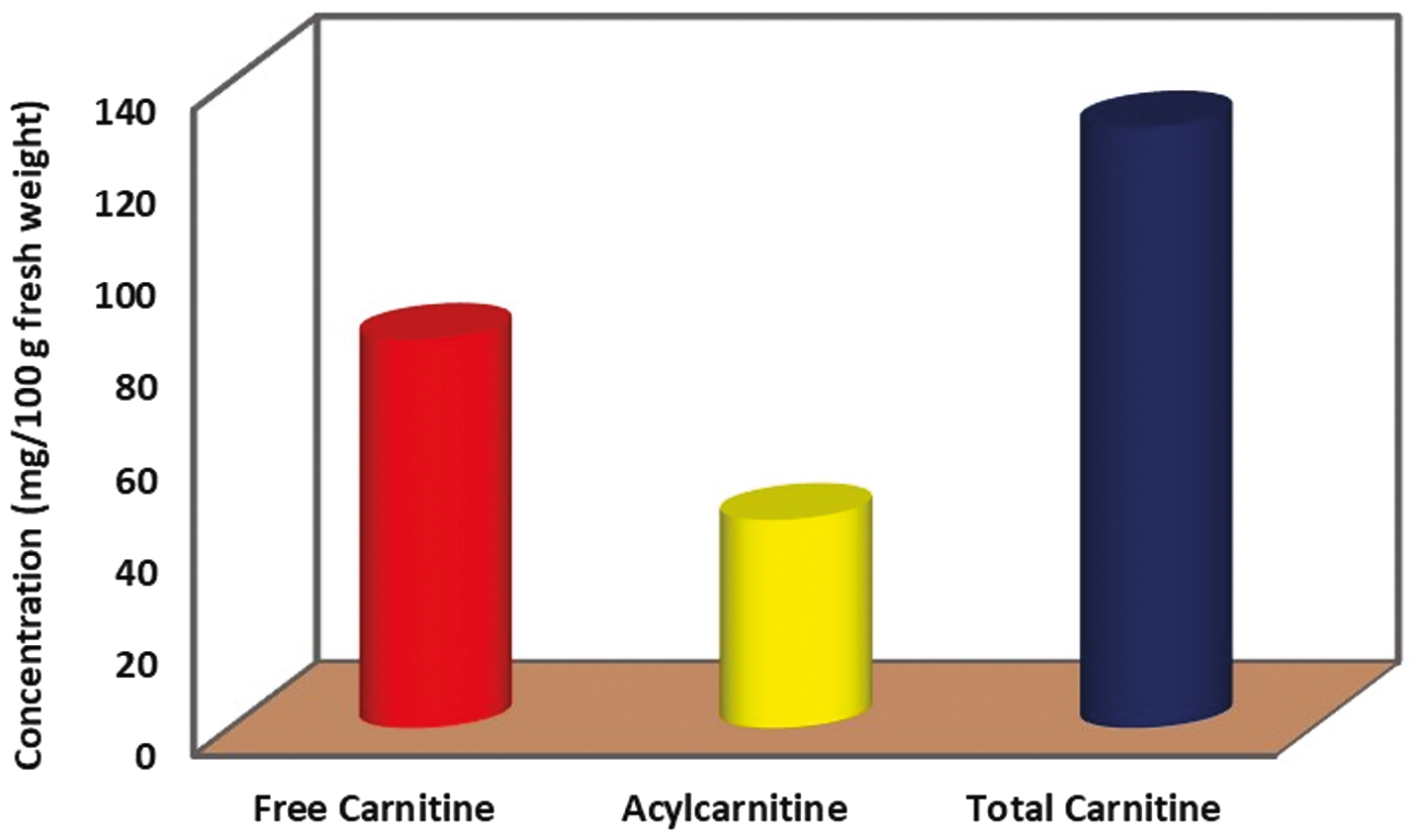
Average concentrations of free carnitine, acylcarnitine, and total carnitine in camel muscle tissue. (Alhomida et al., 1995).

L-carnitine (beta-hydroxy-gamma-trimethyl amino butyric acid) plays an important physiological role in producing energy during exercise through transporting long-chain fatty acids across the inner mitochondrial membranes. Alhomida et al. (1995) reported 5.17, 2.60, and 7.77 µmol/g fresh weight of free carnitine, acylcarnitine, and total carnitine, respectively, in camel meat (Figure 3). While the significance of the concentration cannot be objectively determined as these results have been generated from different laboratories, it is possible that camel meat could potentially be one of the best sources of L-carnitine after goat meat (11.36 µmol/g fresh weight) (Shimada et al., 2004).

## Conclusion

The amino acid and mineral contents of camel meat are often higher than other meat animals, probably due to lower intramuscular fat levels. According to the nutritional values of camel meat, it can be successfully marketed alongside other livestock. Camel meat is low in fat and cholesterol in comparison to other red meat products, which makes it a preferred choice of meat for health-conscious consumers. With the increasing demand for high-protein and low-fat meat products, camel meat will be a suitable product for international markets. Camel meat quality as well as shelf life can be improved by using various pretreatments such as the use of polyphenolics, curing, aging, and packaging. Future research is needed for exploiting the potential of the camel as a source of meat through multidisplinary research into efficient production systems, improved meat technology, and in marketing. It is important to encourage the consumption of camel meat and to devise a national plan to raise awareness among the public due to its nutritional values and uses at a time when the demand for healthy food is greater than ever.

## About the Authors

**Isam T. Kadim** is a professor in the Department of Biological Sciences and Chemistry, College of Arts and Sciences at University of Nizwa, Sultanate of Oman. He obtained his Ph.D. from Massey University, New Zealand. He has more than 40 years of teaching and research experience at four different international institutes. He has supervised many M.Sc. and Ph.D. students; published more than 120 refereed papers, four books, and 17 book chapters; and presented 85 papers at scientific conferences. Muscle biology is a major focus of his research and publications. He has obtained more than US$1.4 million funding for his research projects.

**Issa Al-Amri** is an Associate Professor at the Department of Biological Sciences and Chemistry, and Dean of College of Arts and Sciences at the University of Nizwa, Oman. He received his BSc in Biomedical Sciences form Glasgow Caledonian University, MSc in Biological Electron Microscopy form the University of Wales, Aberystwyth, and PhD in Biological Sciences from the University of Portsmouth in the UK. Dr. Al-Amri worked at the Department of Pathology, College of Medicine & Health Sciences, Sultan Qaboos University at the capacity of Director of Diagnostic Electron Microscopy Unit for more than 24 years before joining the University of Nizwa in 2012. He published more than 110 articles in international peer-reviewed journals and conferences. Dr. Al-Amri experience and research interests includes histopathology, stress physiology, reproductive biology and endocrinology, biotechnology, nanotechnology, electron microscopy and microanalyses.

**Abdulaziz Y. AlKindi** is a Professor of Physiology at the Department of Biological Sciences and Chemistry, Vice Chancellor of Academic Affairs and the Secretary General of the Boards of Trustees at the University of Nizwa, Oman. He received his BSc and MSc from the University of Arizona, USA and his PhD from the University of Exeter in the UK. Prof. AlKindi worked as an Associate Professor at the Department of Biology and Dean of College of Science at Sultan Qaboos University between 1988 and 2009. He published more than 90 articles in international peer-reviewed journals and conferences. Prof. AlKindi main research interests focused on stress and reproductive physiology and endocrinology.

**Q.M. Imranul Haq** is currently working as an Associate Professor in the Department of Biological Sciences and Chemistry, College of Arts and Sciences, at University of Nizwa, Sultanate of Oman. He was awarded Ph.D. in Biotechnology in 2009 from Jamia Millia Islamia, New Delhi, India (research work carried out at IARI, New Delhi). He has 10 years of teaching and 16 years of research experience in the field of Biotechnlogy, Microbiology (Plant Molecular Virology) and Molecular Biology. He has post-doc from The Catholic University of Korea (South Korea), ICGEB (India), and Visiting Research Scientist at University of Arizona (USA). He has published several research papers in different peer-reviewed journals.
